# Hidden Cardiovascular Risk: Early Menopause Triggering Acute Coronary Syndrome (ACS) in Young Patients

**DOI:** 10.7759/cureus.103193

**Published:** 2026-02-08

**Authors:** Jehandad Khan, Sarmad Raza, Salman Nishtar, Noorul Hadi, Muhammad Abdur Rauf, Amna Saleem

**Affiliations:** 1 Cardiology, Kettering General Hospital, Kettering, GBR; 2 General Internal Medicine, Scunthorpe General Hospital, Scunthorpe, GBR; 3 Cardiology, Medical Teaching Institution Mardan Medical Complex, Mardan, PAK; 4 Cardiology, Kuwait Teaching Hospital/Peshawar Medical College, Peshawar, PAK

**Keywords:** acute coronary syndrome, cardiovascular risk, early menopause, nstemi, women’s health

## Abstract

Introduction

Acute coronary syndrome (ACS) primarily affects individuals over 50 years of age. However, younger adults, particularly women experiencing early menopause, represent a unique demographic with significant implications for cardiovascular outcomes. Early menopause, characterized by cessation of menstruation before age 45, has been linked to increased metabolic and cardiovascular risks. This study explores the prevalence of early menopause and its associations with ACS among women aged 35-45 years.

Objective

This study aims to explore the occurrence of early menopause in young women presenting with ACS and examine its associations with cardiovascular risk factors, in-hospital outcomes (including arrhythmias, heart failure, and length of hospital stay), and hormonal parameters (follicle-stimulating hormone (FSH) levels).

Methods

This cross-sectional study was conducted at the Department of Cardiology at Mardan Medical Complex from March to September 2022. A total of 142 women aged 35-45 years with a confirmed diagnosis of ACS, classified as ST-elevation myocardial infarction (STEMI), non-ST-elevation myocardial infarction (NSTEMI), or unstable angina based on clinical presentation, electrocardiographic changes, and cardiac troponin-I levels, were included. Early menopause was defined by ≥12 months of amenorrhea before age 45, corroborated by serum FSH levels >40 mIU/mL measured using standard immunoassay techniques. Women with diabetes mellitus, hypertension, smoking history, hormonal replacement therapy use, chronic systemic disease, or malignancy were excluded. Associations were evaluated using independent t-tests, chi-square tests, and multivariable logistic regression, adjusting for potential confounders.

Results

Early menopause was identified in 36 participants (25.4%). Women with early menopause had a significantly higher body mass index compared to non-menopausal women (mean difference: 1.6 kg/m²; p = 0.02). Mean FSH levels were markedly elevated in the early menopause group (67.8 ± 6.4 vs. 16.4 ± 4.3 mIU/mL; p < 0.01), with the highest levels observed among NSTEMI patients. Early menopausal women experienced longer hospital stays (mean difference: 0.8 days; p = 0.03) and higher odds of in-hospital arrhythmias and heart failure, indicating increased vulnerability to adverse short-term outcomes.

Conclusion

Early menopause is common among young women presenting with ACS and is associated with unfavorable clinical characteristics and in-hospital outcomes. These findings suggest that early menopause may act as an underrecognized cardiovascular risk enhancer in younger women. The absence of long-term follow-up represents a limitation and warrants future prospective studies to determine its impact on long-term cardiovascular outcomes.

## Introduction

Acute Coronary Syndrome (ACS) represents a spectrum of clinical conditions caused by an acute reduction in myocardial blood flow and includes unstable angina, non-ST-elevation myocardial infarction (NSTEMI), and ST-elevation myocardial infarction (STEMI) [1]. Although ACS predominantly affects older adults, recent clinical observations and epidemiological trends indicate a rising burden among younger individuals, particularly women under 45 years of age, who often present with atypical symptoms and delayed diagnosis [2]. In South Asian populations, including Pakistan, premature cardiovascular disease is increasingly reported, with women experiencing coronary events at a younger age compared to Western cohorts, underscoring the need for focused regional investigations.

The relative protection conferred by endogenous estrogen in premenopausal women diminishes with menopause, resulting in increased susceptibility to ischemic heart disease [3]. Estrogen deficiency adversely affects vascular homeostasis through impaired endothelial nitric oxide production, increased vascular inflammation, oxidative stress, and altered lipid metabolism, all of which contribute to endothelial dysfunction and plaque instability, key mechanisms in the pathogenesis of ACS.

Early menopause, defined as permanent cessation of menstruation before the age of 45 years, may occur naturally or following surgical oophorectomy and is associated with adverse metabolic changes such as dyslipidemia, hypertension, central obesity, and insulin resistance [4,5]. These changes accelerate atherosclerotic processes and heighten cardiovascular risk. Despite growing recognition of early menopause as a cardiovascular risk enhancer, data describing its prevalence, clinical profile, and impact on ACS presentation and outcomes in younger women remain limited, particularly in low- and middle-income countries [6].

Notably, most existing studies have focused on postmenopausal or older women, leaving a critical knowledge gap regarding the role of early menopause in women aged 35-45 years presenting with ACS. The lack of region-specific data and limited exploration of hormonal biomarkers further restrict effective risk stratification in this population.

Therefore, this study aims to investigate the occurrence of early menopause in younger women with ACS and evaluate its association with cardiovascular risk factors, ACS subtypes, and short-term clinical outcomes. By addressing this underexplored population, the study seeks to highlight early menopause as a potential non-traditional and often overlooked risk enhancer, thereby supporting more individualized cardiovascular risk assessment and management strategies in young women.

## Materials and methods

Study design and setting

This retrospective cross-sectional study was conducted in the Department of Cardiology at Mardan Medical Complex over a six-month period from March 2022 to September 2022. Ethical approval was obtained from the Institutional Review Board of Medical Teaching Institution Bacha Khan Medical College, Mardan, prior to study initiation (IRB approval no. 187/BKMC).

Eligible participants were enrolled using a consecutive sampling method, whereby all women who met the inclusion criteria and presented during the study period were invited to participate. This approach was adopted to minimize selection bias and enhance internal validity.

Study population

A total of 142 female patients aged 35-45 years presenting with a confirmed diagnosis of ACS were included. Written informed consent was obtained from all participants prior to enrollment. Inclusion criteria consisted of women within the specified age range with a final diagnosis of ACS. ACS was classified into STEMI, NSTEMI, or unstable angina.

Women were excluded if they had established conventional cardiovascular risk factors, including hypertension, diabetes mellitus, active or prior smoking, or a strong family history of premature cardiovascular disease. This exclusion was intentional to reduce confounding and to isolate the independent association between early menopause and ACS in younger women. Additional exclusion criteria included prior use of hormone replacement therapy or oral contraceptive pills within the preceding six months, known familial dyslipidemias, malignancy, thromboembolic disorders, stroke, chronic liver or kidney disease, chronic obstructive pulmonary disease, asthma, or established heart failure.

Data collection

Data were collected through structured face-to-face interviews and review of hospital medical records using a standardized proforma. Demographic variables included age and BMI.

ACS diagnosis and subtype classification were based on clinical presentation, electrocardiographic findings, and cardiac biomarker elevation (troponin-I levels), in accordance with standard diagnostic criteria. STEMI was defined by persistent ST-segment elevation with positive cardiac biomarkers, NSTEMI by elevated troponin levels without ST-segment elevation, and unstable angina by ischemic symptoms with ECG changes in the absence of biomarker elevation.

Blood samples for FSH measurement were obtained in the fasting state during morning hours and analyzed using a quantitative multiplex immunoassay on the BIO-RAD Bio-Plex® 200 Analyzer System (Bio-Rad Laboratories, Hercules, CA, USA), following the manufacturer’s instructions. All analyses were performed by trained laboratory personnel under the supervision of qualified pathologists, and the laboratory reference range for postmenopausal FSH was >40 mIU/mL.

Laboratory parameters, including lipid profile and fasting blood glucose, were recorded. In-hospital clinical outcomes such as length of hospital stay, development of arrhythmias, and occurrence of heart failure were documented prospectively during admission.

Statistical analysis

Data were analyzed using IBM SPSS Statistics for Windows, Version 25 (Released 2017; IBM Corp., Armonk, New York). Continuous variables were expressed as mean ± SD, and categorical variables were presented as frequencies and percentages. Group comparisons were performed using independent t-tests for continuous variables and chi-square tests for categorical variables. Missing data were minimal (<5%) and were handled using complete-case analysis. No formal sample size or power calculation was performed due to the exploratory nature of the study. Multivariable logistic regression analyses were conducted to assess independent associations between early menopause and clinical outcomes, explicitly adjusting for potential confounders including age and BMI. Additional variables considered in the models, based on known relationships with menopause and cardiovascular risk, included lipid profile and fasting blood glucose. Effect estimates were expressed as OR with 95% CI. A p-value < 0.05 was considered statistically significant.

## Results

Among the 142 enrolled female participants, the mean age was 39.25 ± 3.14 years. Out of these, 36 (25.4%) were identified as having early menopause, while 106 (74.6%) were not menopausal.

Table 1 outlines the baseline characteristics of the study population. Women with early menopause had a significantly higher mean BMI (28.4 ± 3.2 kg/m²) compared to those without menopause (26.8 ± 3.0 kg/m²; p=0.02).

**Table 1:** Participant characteristics Continuous variables are expressed as mean ± SD, and categorical variables as n(%). Odds ratios (OR) are unadjusted. P-values < 0.05 were considered statistically significant. Baseline characteristics were used to adjust multivariable models for clinical outcomes. ACS: acute coronary syndrome; STEMI: ST-elevation myocardial infarction; NSTEMI: non-ST-elevation myocardial infarction; CI: confidence interval

Characteristic		Early Menopause (n = 36)	Non-Menopause (n = 106)	p-value	95% CI/Effect Size
Mean Age (years)		39.3 ± 3.1	39.3 ± 3.1	-	-
Mean BMI (kg/m²)		28.4 ± 3.2	26.8 ± 3.0	0.02	0.3–2.9
ACS Subtype	STEMI	12 (33.3%)	40 (37.7%)	0.62	OR 0.8 (0.4–1.7)
	NSTEMI	15 (41.7%)	39 (36.8%)	0.59	OR 1.2 (0.6–2.4)
	Unstable Angina	9 (25.0%)	27 (25.5%)	0.95	OR 0.98 (0.4–2.3)

Mean FSH levels were significantly elevated in early menopausal women (67.8 ± 6.4 mIU/mL) compared to their non-menopausal counterparts (16.4 ± 4.3 mIU/mL, p < 0.01). These FSH levels in early menopausal women with ACS consistently exceeded the general menopausal threshold (40-60 mIU/mL). The highest levels were observed in NSTEMI patients (68.9 ± 5.8 mIU/mL), followed by STEMI (65.1 ± 6.2 mIU/mL) and unstable angina (63.7 ± 4.9 mIU/mL, p = 0.02). Details of FSH levels according to ACS subtypes are provided in Table 2.

**Table 2:** FSH levels and early menopause Reference range for menopausal status: 40–60 mIU/mL. P-values indicate comparisons across ACS subtypes in early menopausal women; p<0.05 is considered statistically significant. FSH: follicle-stimulating hormone; ACS: acute coronary syndrome; STEMI: ST-elevation myocardial infarction; NSTEMI: non-ST-elevation myocardial infarction

Condition	Early Menopause (n = 36)	Non-Menopause (n = 106)	p-value
Mean FSH (mIU/mL)	67.8 ± 6.4	16.4 ± 4.3	<0.01
FSH Levels by ACS Subtype			
NSTEMI	68.9 ± 5.8	-	0.02
STEMI	65.1 ± 6.2	-	-
Unstable Angina	63.7 ± 4.9	-	-

Table 3 presents clinical outcomes between the two groups. Women with early menopause had a significantly longer hospital stay (4.5 ± 1.2 days) compared to non-menopausal women (3.7 ± 1.0 days, p=0.03). The rate of in-hospital arrhythmias was higher in early menopausal women (7, 19.4%) than in non-menopausal women (12, 11.3%; p=0.05). Similarly, heart failure developed in 4 (11.1%) of early menopausal patients compared to 6 (5.7%) in the non-menopausal group (p=0.07).

**Table 3:** Clinical outcomes ^†^Small number of events limits statistical power; results should be interpreted as trends. Multivariable logistic regression adjusted for age and BMI. Continuous outcomes are expressed as mean ± SD, and categorical outcomes as n (%). Adjusted β represents the difference in hospital stay; adjusted OR represents the odds of the event. Missing data <5% were handled using complete-case analysis. A p-value < 0.05 is considered statistically significant. BMI: body mass index; CI: confidence interval; OR: odds ratio

Outcome	Early Menopause (n = 36)	Non-Menopause (n = 106)	Adjusted Effect (95% CI)	p-value
Hospital Stay (days)	4.5 ± 1.2	3.7 ± 1.0	Adjusted β = 0.8 days (0.1–1.5)	0.03
In-hospital Arrhythmias (%)	7 (19.4%)	12 (11.3%)	Adjusted OR 1.9 (0.8–4.5)^†^	0.05
In-hospital Heart Failure (%)	4 (11.1%)	6 (5.7%)	Adjusted OR 2.1 (0.6–7.2)^†^	0.07

## Discussion

This study provides significant evidence of the prevalence and clinical implications of early menopause among women presenting with ACS. The prevalence of 25.4% observed in our cohort aligns with previous studies, highlighting the importance of clinical awareness regarding the potential impact of early menopause on cardiovascular health [7,8]. Early menopause, typically defined as menopause occurring before 45 years of age, is increasingly recognized as a significant risk for coronary artery disease [9]. This study adds to the existing literature, showcasing that women with early menopause represent a unique, high-risk population within the ACS cohort, particularly in South Asian settings where cardiovascular disease occurs at a younger age. Evidence from Pakistan supports this observation; Ali et al. reported that women with premature or early menopause had a significantly higher burden of cardiovascular risk factors and earlier presentation of ischemic heart disease compared to age-matched premenopausal women, underscoring the regional relevance of our findings [10].

In our study, early menopause was identified in 25.4% of women with ACS, which is notably higher than the general population's prevalence of early menopause [10,11]. This emphasizes the need to consider early menopause as an independent risk factor for the assessment of cardiovascular health in women. Large cohort data from the United States, including the Multi‑Ethnic Study of Atherosclerosis, have demonstrated that early menopause is associated with a significantly increased risk of future coronary heart disease and stroke, independent of traditional cardiovascular risk factors, with adjusted hazard ratios of approximately 2.08 and 2.19, respectively, supporting the biological plausibility of our results [12]. Similar associations have been reported in international cohorts from Europe and East Asia, where early menopause has been linked to a 1.5-2-fold increase in coronary events, supporting the external relevance of our findings. The demographic profile of early menopausal women in our cohort revealed a higher mean BMI (28.4 ± 3.2 vs. 26.8 ± 3.0, p = 0.02) compared to their non-menopausal counterparts. The elevated BMI could be a contributing factor for other established risk factors for coronary artery disease, such as hypertension, dyslipidemia, and insulin resistance, which are commonly seen in this group [11,13]. Although lifestyle factors, dietary habits, and socioeconomic status were not directly assessed, these variables may influence both menopausal timing and cardiovascular risk and represent potential residual confounders.

The pathophysiology of cardiovascular disease in early menopause is closely linked to estrogen deficiency [12]. The loss of estrogen accelerates atherogenesis through multiple mechanisms, including lipid abnormalities, endothelial dysfunction, and pro-inflammatory states [13-15]. Estrogen has a protective effect on vascular endothelial cells, contributing to improved endothelial function and reduced inflammation [15,16]. Therefore, the premature decrease in estrogen in early menopause leads to a heightened risk of cardiovascular events, including ACS. Additionally, estrogen deficiency has been associated with autonomic imbalance, increased arterial stiffness, and impaired coronary vasoreactivity, all of which may predispose to ischemic events even in the absence of obstructive coronary disease [17,18].

Our study further supports this hypothesis by demonstrating significantly elevated FSH levels in early menopausal women (67.8 ± 6.4 mIU/mL vs. 16.4 ± 4.3 mIU/mL, p < 0.01), consistent with the known role of estrogen deficiency in cardiovascular pathology. FSH levels, a marker of ovarian function, are elevated when estrogen levels decline, reflecting the severity of hormonal imbalance [19]. Emerging evidence suggests that FSH itself may exert direct vascular effects by promoting endothelial inflammation, oxidative stress, and adverse lipid metabolism, thereby contributing independently to cardiovascular dysfunction [20,21]. In this study, FSH levels in early menopausal women with ACS exceeded the general menopausal threshold of 40-60 mIU/mL, with the highest levels observed in NSTEMI patients (68.9 ± 5.8 mIU/mL). This finding may reflect a predominance of coronary microvascular dysfunction or supply-demand mismatch in NSTEMI, conditions that are particularly sensitive to hormonal deprivation, rather than acute plaque rupture alone [16,22-23]. The findings highlight the need for more personalized diagnostic approaches, including advanced imaging techniques, to assess coronary microvascular dysfunction in early menopausal women.

Limitations

This study has several limitations that should be considered when interpreting the findings. First, its single-center, cross-sectional design limits external generalizability and precludes causal inference; the observed associations cannot establish that early menopause directly causes ACS. Second, the modest overall sample size, particularly the smaller subgroup of women with early menopause (n = 36), reduced statistical power for subgroup analyses and may account for borderline associations observed for certain in-hospital outcomes, such as arrhythmias and heart failure. Third, the exclusion of women with conventional cardiovascular risk factors (hypertension, diabetes, smoking, strong family history) was intended to strengthen internal validity and isolate the independent association between early menopause and ACS; however, this approach may introduce selection bias and limit applicability to the broader, real-world ACS population in which these risk factors frequently coexist. Fourth, potential confounders, including lifestyle behaviors, dietary patterns, physical activity, hormonal therapy use, and socioeconomic status, were not assessed and may influence both menopausal timing and cardiovascular risk, representing residual confounding. Fifth, the absence of longitudinal follow-up prevents evaluation of long-term cardiovascular outcomes, including recurrent ACS or mortality. Finally, while multivariable analyses adjusted for age and BMI, other unmeasured biological or environmental factors may affect the observed relationships. Larger, multicenter prospective studies with comprehensive adjustment for biological, lifestyle, and socioeconomic variables are warranted to validate these findings and clarify the mechanistic pathways linking early menopause to cardiovascular disease.

## Conclusions

This study highlights the association between early menopause and adverse clinical profiles among younger women presenting with ACS. The high prevalence of early menopause observed in our cohort, together with elevated FSH levels and less favorable in-hospital outcomes, underscores the importance of heightened clinical awareness of menopausal status in women with ACS. Although early menopause itself is not directly reversible, its associated cardiovascular risk may be mitigated through targeted lifestyle modification, optimization of cardiometabolic risk factors, and selected pharmacologic or hormonal strategies; however, the observational, cross-sectional design of this study precludes causal inference.

In clinical practice, incorporating menopausal assessment into ACS evaluation may be operationalized through routine documentation of age at menopause, consideration of biochemical confirmation (e.g., FSH measurement) when clinically appropriate, and integration of menopausal status into cardiovascular risk stratification frameworks. Future research should prioritize longitudinal cohort studies and mechanistic investigations to validate these associations, clarify underlying biological pathways, and assess whether targeted preventive or therapeutic interventions can improve outcomes. Early identification of women with premature or early menopause may enable more personalized cardiovascular prevention strategies and closer surveillance in this potentially vulnerable population.
